# Effluxosomes and the evolution of metal resistance in *Mycobacterium tuberculosis*

**DOI:** 10.1128/iai.00307-26

**Published:** 2026-06-26

**Authors:** Louis Benastre, Pierre Dupuy, Claude Gutierrez, Pascal Demange, Olivier Neyrolles

**Affiliations:** 1 CNRS, IPBS, University of Toulouse, Toulouse, France; University of California Davis, Davis, California, USA

**Keywords:** effluxosomes, P-type ATPases, metal detoxification, *Mycobacterium tuberculosis*, host-pathogen interactions

## Abstract

During infection, host immune cells deploy a variety of strategies to neutralize invading pathogens, including the manipulation of metal availability, a process traditionally understood as nutritional immunity. While depriving microbes of essential metals, such as iron and manganese, inhibits their growth, host cells also engage in metal intoxication, actively overloading phagosomes with toxic levels of transition metals, such as copper and zinc. To survive these dual pressures, *Mycobacterium tuberculosis*, the etiological agent of tuberculosis, has evolved specialized metal resistance mechanisms. This review explores how *M. tuberculosis* counters host-imposed metal stress through an arsenal of P-type ATPases, particularly the diverse P_1B_ subfamily of transition metal exporters. We detail the structural features, metal specificities, and regulatory mechanisms of *M. tuberculosis*’ 12 P-type ATPases, focusing on three key systems, CtpC, CtpG, and CtpV, and their cognate scaffold proteins PacL1, PacL2, and PacL3. These PacL-Ctp pairs form dynamic membrane assemblies termed effluxosomes, which mediate resistance to transition metals such as zinc and cadmium. The review also highlights several distinctive features of *M. tuberculosis* P_1B_-ATPases relative to canonical transporters such as CopA and ZntA, suggesting unique adaptations to the intracellular environment. Finally, we discuss the challenges of functionally and structurally characterizing these systems and propose future directions to elucidate effluxosome assembly and function. Together, these insights reveal how *M. tuberculosis* leverages metal export as a critical survival strategy and suggest novel therapeutic opportunities targeting metal detoxification pathways.

## METAL STRESS AS A HOST DEFENSE

The virulence and persistence of prokaryotic pathogens during infection critically depend on their capacity to evade and resist host immune defenses. Once detected, invading bacteria must be rapidly neutralized to prevent infection and disease progression, a task largely carried out by professional phagocytes such as macrophages. Within these immune cells, pathogens are exposed to a hostile environment that includes potent antimicrobial mechanisms such as phagosome acidification and the generation of reactive oxygen and nitrogen species (1). In parallel, host cells impose additional stress by limiting the availability of essential metal ions through a process known as nutritional immunity (2–6). This strategy deprives pathogens of vital micronutrients, most notably iron and manganese, often via metal transporters like NRAMP1, thereby inhibiting microbial growth and survival (7). Among human pathogens, *Mycobacterium tuberculosis*, the causative agent of tuberculosis (TB) and the leading infectious killer worldwide, has developed highly specialized mechanisms to withstand these immune pressures. In particular, *Bacillus* has evolved multiple strategies to overcome metal starvation. These include the deployment of high-affinity iron acquisition systems, such as the synthesis of siderophores (mycobactins and carboxymycobactins) (8, 9), as well as the ability to utilize host-derived heme as an alternative iron source (10, 11). This ongoing tug-of-war over metal resources represents a critical front in the battle between the host and the pathogen, shaping both the pathogenesis and persistence of *M. tuberculosis* within the host.

Building on the concept of nutritional immunity, more recent research has uncovered a complementary host defense strategy: metal intoxication. Rather than depriving pathogens of essential metals, macrophages can actively accumulate transition metals, such as zinc and copper, to toxic levels within phagosomes (12, 13). This process disrupts bacterial metal homeostasis, damages essential proteins, and amplifies oxidative stress (13–16). Although trace amounts of these metals are vital for both host and pathogen physiology, excessive concentrations, especially of redox-active metals, can catalyze Fenton and Haber-Weiss reactions, generating reactive oxygen species that damage DNA, proteins, and lipids. In addition, elevated metal levels can lead to mis-metalation, where incorrect metal ions displace native cofactors in metalloenzymes, impairing their structure and function. These toxic effects are exploited by the host to combat intracellular pathogens, including *M. tuberculosis*. To counteract these host-imposed stresses, bacteria deploy intricate homeostatic systems comprising metal-sensing regulators, trafficking pathways, and assembly of machineries that collectively preserve metal balance and support essential metalloprotein function (17). Interestingly, clinical observations have reported systemic zinc deficiency in individuals with TB, raising the hypothesis that insufficient zinc availability may impair host immune function and compromise the ability to control infection (18–20).

To withstand the toxic effects of metal overload imposed by the host, *M. tuberculosis* has evolved specialized metal export systems that enable it to resist metal intoxication and survive within macrophages. This detoxification capacity is believed to be critical for the long-term persistence of the pathogen and for granuloma formation during chronic infection. Genomic analyses of *M. tuberculosis* have revealed two major classes of putative metal efflux transporters: a single cation diffusion facilitator and 12 P-type ATPases (21, 22). Among these 12, 7 belong to the P_1B_-type ATPase subfamily, transporters specialized in the export of transition metals, highlighting their likely role in enabling *M. tuberculosis* to adapt to the metal-rich environment of the host phagosome. Additional putative transport systems, including ABC importers and ZIP- or NiCoT-family transporters, may also contribute to metal homeostasis in *M. tuberculosis*, although their precise roles remain to be experimentally established (23–26).

## P-TYPE ATPASES: GENERAL MECHANISM AND STRUCTURAL FEATURES

**Fig 1:**
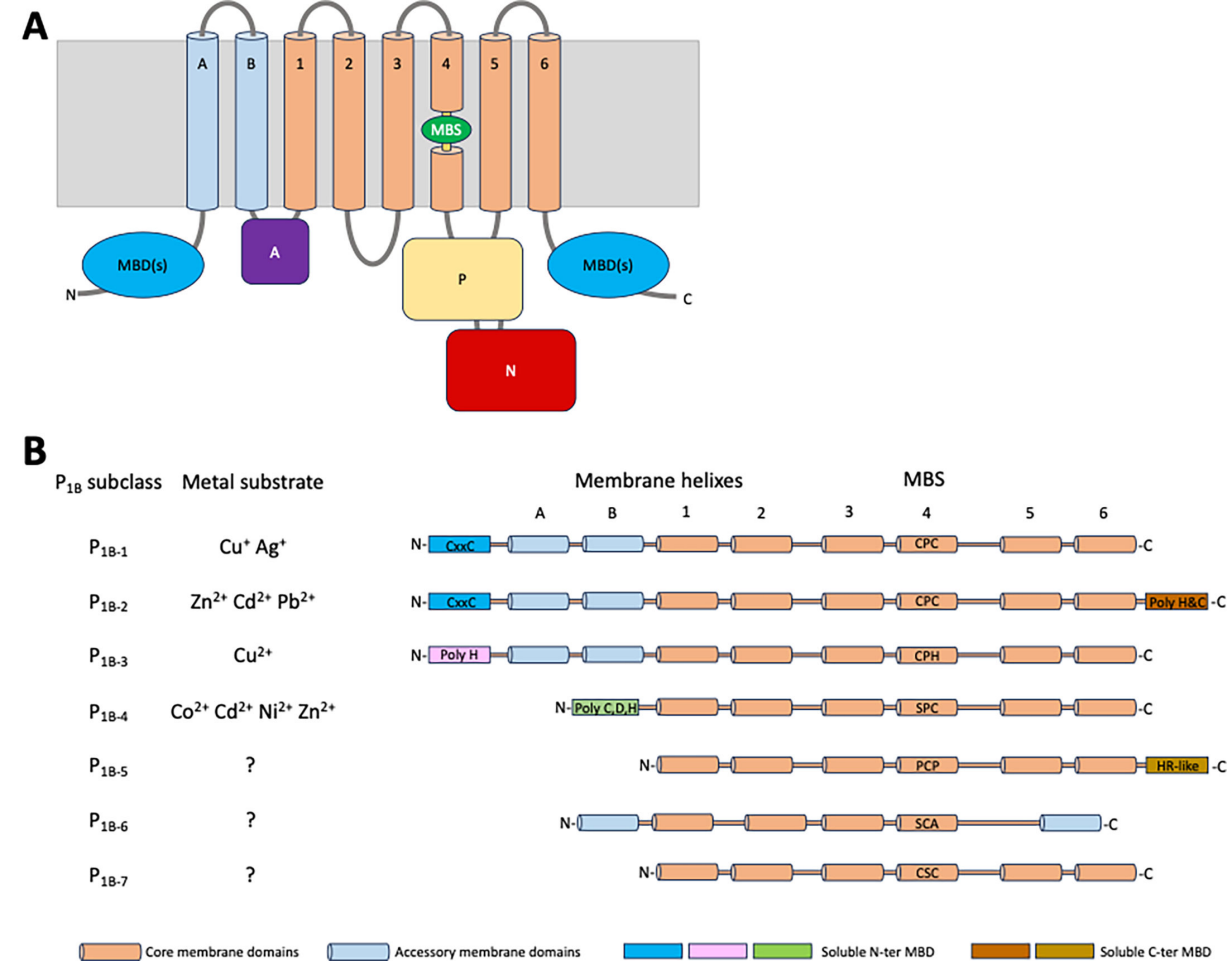
Structural organization, membrane topology, and metal specificity of P_1B_-ATPases. (**A**) Schematic representation of a typical P_1B_-ATPase embedded in the plasma membrane. Its catalytic core consists of the soluble actuator (A), phosphorylation (P), and nucleotide-binding (N) domains. Conserved residues within the fourth transmembrane helix (TM4) constitute the metal-binding site (MBS), while cytosolic metal-binding domains (MBDs) are proposed to play important regulatory roles. (**B**) Topology and metal specificity of P_1B_-ATPases, as described in previous studies (38, 23, 27, 33). Schematic comparison of the membrane topology of different P_1B_-ATPase subclasses and their associated metal substrates. All P_1B_-ATPases contain 6–8 transmembrane helices (cylinders) and a conserved MBS. Depending on the subclass, these enzymes may include one or more soluble N- and/or C-MBDs (shown as rectangles), often featuring subclass-specific amino acid motifs.

P-type ATPases are a large and functionally diverse family of membrane proteins involved in the active transport of various substrates, including lipids and ions such as calcium, sodium, potassium, protons, and transition metals. Members of the P5 subfamily have also been proposed to transport polyamines and, possibly, hydrophobic transmembrane α-helices or misinserted membrane segments (27, 28). Despite their substrate diversity, all P-type ATPases share a conserved catalytic mechanism based on ATP hydrolysis and the transient phosphorylation of a conserved aspartate residue within the highly conserved DKTG motif (27, 29–31). This mechanism is supported by a characteristic core structure comprising four distinct domains (Fig. 1A): three cytosolic domains (actuator [A], phosphorylation [P], and nucleotide-binding [N]), and a transmembrane (T) region (30, 32, 33). These enzymes operate through the Post-Albers cycle, classically described as a series of transitions between the major states E_1_ → E_1_P → E_2_P → E_2_ (34, 35). In the E1 state, the enzyme binds substrate on the cytosolic side and ATP-driven phosphorylation of the conserved aspartate in the P domain generates the E1P intermediate (34). The subsequent E1P-to-E2P transition produces the outward-facing conformation and is coupled to substrate release to the opposite side of the membrane. Dephosphorylation then occurs from the E2P state, with the conserved glutamate of the A-domain catalyzing hydrolysis of the aspartyl phosphate. In many P-type ATPases, this step is promoted by the occlusion of a counter-transported ion, whereas in P_1B_-ATPases, such a counter-ion is not clearly established. The enzyme then relaxes through E2 back to E1, completing the transport cycle (36, 37). Despite important mechanistic variations among subclasses, this overall reaction cycle underlies the functional versatility and essential biological roles of P-type ATPases across all domains of life.

## P_1B_-ATPASES: SUBCLASSES AND STRUCTURAL DIVERSITY

Among the various subclasses of P-type ATPases, the P_1B_ subfamily is responsible for the transport of transition metal ions (Fig. 1B). These ATPases mediate the translocation of both essential and toxic metals, including Cu^+^, Zn^2+^, Pb^2+^, Fe^2+^, Co^2+^, Cd^2+^, and Mn^2+^, and are therefore central to cellular metal homeostasis and detoxification processes (38). Historically, the best-characterized members of this subfamily are the eukaryotic copper-transporting ATPases ATP7A and ATP7B, whose dysfunction is linked to severe human diseases. Mutations in ATP7A, also known as the Menkes protein, lead to systemic copper deficiency, causing progressive neurodegeneration and early death in affected children (39, 40). Mutations in ATP7B (responsible for Wilson’s disease) result in toxic copper accumulation in tissues, leading to hepatic and neurological symptoms (41, 42).

The association between these genes and human disease was established in the early 1990s, around the same time that the term “heavy metal P-type ATPases” gained traction in the literature. Before this, references to such ATPases were rare; for example, CadA was one of the first heavy metal ATPases described in bacteria by Nucifora et al. in 1989 (43). The term “P_1_-ATPase” was formally introduced in 1995 to distinguish ATPases that transport heavy metals from the P_2_ subfamily, which includes transporters of more common ions like Na^+^, K^+^, and Ca^2+^ (44). Recent insights suggest that P_1B_-ATPases constitute the most structurally and functionally diverse group within the P-type family, likely reflecting long evolutionary adaptation to a wide array of metal substrates. This diversity, however, also complicates their classification and functional characterization.

Like all P-type ATPases, P_1B_-ATPases share a conserved catalytic architecture comprising three cytosolic domains, actuator (A), P, and nucleotide-binding (N), and a membrane-embedded region responsible for substrate translocation. However, P_1B_-ATPases possess several unique structural features that distinguish them from other P-type subfamilies. P_1B_-ATPases typically contain eight TM helices. The six helices M1–M6 form the canonical core membrane domain found in most P-type ATPases, whereas two additional helices, MA and MB, are specific to many P_1B_-ATPases. These auxiliary helices are not yet fully understood but may contribute to metal specificity or regulation (45).

A hallmark of P_1B_-ATPases is the presence of one or more metal-binding domains (MBDs) located at the N- or C-terminal ends. These domains are usually rich in cysteine and histidine residues and can adopt a ferredoxin-like fold, a common structural motif among metal-binding proteins (46). In addition to terminal MBDs, P_1B_-ATPases also contain conserved motifs within their transmembrane helices, particularly the cysteine-containing tripeptide motif in TM4, which constitutes the transmembrane MBS. Based on their metal substrate preferences, the nature of their MBDs, and the MBS sequence, P_1B_-ATPases are classified into seven subtypes (P1_B-1_–P1_B-7_) (38, 46, 47). For example, P_1B-2_-ATPases, characterized by the CPC motif in TM4, typically transport Zn**^2+^**, Cd**^2+^**, and Pb**^2+^** (48–50), while P_1B-4_-ATPases, marked by an SPC motif, transport Co**^2+^**, Cd**^2+^**, Ni^2+^, and Zn**^2+^** (51–55). The metal specificity of other subtypes remains less defined: P_1B-5_-ATPases (with a PCP motif) have been linked to Ni^2+^ and Fe^2+^ transport, and P_1B-6_- and P_1B-7_-ATPases, characterized by SCA and CSC motifs, respectively, are still poorly understood (56, 57). The P_1B-3_ subclass, once thought to export Cu^2+^, has been reclassified as part of the P_1B-1_ group after it was confirmed to transport Cu^+^ only (58). Notably, overlaps in substrate handling between subfamilies, such as the shared ability of both P_1B-2_ and P_1B-4_ ATPases to transport Zn^2+^ and Cd^2+^, suggest a degree of functional redundancy that may provide adaptive flexibility under varying metal stress conditions.

Although the full molecular mechanism of P_1B_-ATPases remains to be elucidated, recent studies highlight the regulatory role of their MBDs, suggesting that these domains may modulate transport activity in response to intracellular metal concentrations or interaction with metallochaperones (59). MBDs have also been proposed to directly participate in metal delivery by acquiring metals from donor ligands and transferring them to the TM region for transport (53, 60).

A prime example of the regulatory complexity of MBDs in P_1B_-ATPases comes from the eukaryotic copper-transporting ATPases ATP7A and ATP7B. Both proteins contain six N-terminal MBDs, each approximately 70 amino acids in length, capable of binding reduced Cu^+^ via a conserved CxxC motif. These six domains constitute six distinct MBSs. Although structurally similar, these MBDs play non-redundant functional roles, particularly in ATP7B, which mediates copper transport to the Golgi for the metallation of copper-dependent enzymes (61, 62). Mutational studies have shown that MBS_5_ and MBS_6_ are critical for regulating activity: mutations in these two domains dramatically enhance copper sensitivity (~7-fold to 8-fold), while MBS_1-4_ appear dispensable for activity regulation (63). Structural analyses further support this functional distinction. In recent ATP7B cryoEM structures, MBD_1-4_ are disordered, whereas MBD_5_ and MBD_6_ are well-resolved, closely associating with the first transmembrane segment and the A domain (64, 65). This spatial arrangement suggests that MBD_5-6_ exert a regulatory role, likely involved in autoinhibition of the ATPase.

Additional structural and biochemical evidence supports the model whereby MBDs play a regulatory role. In the absence of copper, MBD_1-3_ interact transiently with one another, and this interaction appears to suppress ATPase activity, as selective proteolysis of MBD_1-3_ relieves this inhibition and activates ATP hydrolysis (66, 67). Furthermore, copper is delivered to these domains by the cytosolic metallochaperone Atx1, and loading of MBD_3_ with Cu^+^ has been shown to release autoinhibition (68).

Together, these findings illustrate the sophisticated regulatory roles of MBDs in modulating the activity of P_1B_-ATPases, functioning not only as passive metal carriers but also as dynamic sensors and regulators of enzymatic function, as suggested for other copper transporters, such as CopA (59, 69, 70).

## CANONICAL BACTERIAL SYSTEMS: COPA AND ZNTA

A well-characterized bacterial P_1B-1_-type ATPase is CopA, originally identified in *Escherichia coli* (71). Subsequent studies demonstrated that *copA* encodes a copper-exporting ATPase, playing a key role in maintaining intracellular copper homeostasis (72). CopA homologs are widely conserved across numerous pathogenic prokaryotes and are frequently associated with virulence, reflecting the importance of copper detoxification for survival within host cells (73). This is consistent with the fact that the immune system uses copper as an antimicrobial weapon, particularly within phagosomes of activated macrophages. Functional studies have implicated CopA in copper tolerance across a range of bacterial species, including *E. coli* (72), *Bacillus subtilis* (74), *Haemophilus influenzae* (75), *Listeria monocytogenes* (76), *Acinetobacter baumannii* (77), *Actinobacillus pleuropneumoniae* (78)*,* and several *Streptococcus* species such as *S. aureus* (79), *S. pneumoniae* (80, 81)*,* and *S. pyogenes* (82). Furthermore, in *A. baumannii* (77), *S. pneumoniae* (80, 81), *S. pyogenes* (82), and *Pseudomonas aeruginosa* (83), CopA-mediated copper export enhances the pathogen’s ability to resist copper toxicity and persist in hostile, immune-activated environments.

CopA exhibits the canonical P_1B_ topology, with eight TM helices, including a CPC motif in TM4, essential for copper translocation. It also contains the three cytosolic A, P, and N catalytic domains, as well as two N-terminal MBDs, each harboring a CxxC motif that confers high-affinity binding to Cu^+^ (69, 84). Interestingly, while these motifs enable copper coordination, they are not essential for copper resistance or transport activity in *E. coli* (85).

During phagocytosis-induced metal stress, *E. coli* relies on CopA to export Cu^+^ ions from the cytoplasm to the periplasm (72, 86). This process is coupled to ATP hydrolysis and may involve transfer of Cu^+^ to the periplasmic chaperone CusF, which subsequently facilitates copper export from the cell (87). In the cytoplasm, CopA interacts with CopZ, a soluble copper chaperone that is uniquely translated via ribosomal frameshifting from the *copA* mRNA (88). This interaction is copper-dependent, with Cu^+^-loaded CopZ exhibiting increased affinity for CopA (89). Mechanistically, Cu^+^-loaded CopZ dimers transiently interact with the N-terminal MBDs of CopA, delivering copper to the ATPase for translocation (90). While MBDs were originally thought to serve as intermediate carriers, directing Cu^+^ from chaperones like CopZ to the TM4 CPC site, growing evidence suggests that their primary role is regulatory, similar to what has been observed in eukaryotic ATPases such as ATP7A (47, 61) and ATP7B (63–68). Supporting this, CopZ cannot activate a truncated CopA lacking MBDs, yet retains the ability to activate CopA variants with MBDs that are unable to bind copper (91). This indicates that copper transfer for transport depends on CopZ, regardless of MBD metal-binding capability. Moreover, this regulatory interaction is biologically significant: CopZ is critical for biofilm formation in *Streptococcus mutans* (92), further underscoring the functional importance of chaperone-mediated copper handling in bacterial physiology. In some bacteria, such as *S. pneumoniae*, the membrane-anchored copper chaperone CupA works alongside CopA, binding Cu^+^ ions via conserved cysteine and methionine residues and facilitating their direct transfer to CopA for efflux (93, 94). CupA is essential for copper resistance under host-imposed copper stress and contributes to pathogen survival within phagocytes (81).

Although the copper-binding properties of CopA MBDs are not required for copper efflux, their structural similarity to copper chaperones like CopZ suggests a possible evolutionary relic rather than a direct functional role. While the membrane chaperone CupA adopts a β-sheet-based cupredoxin fold (94), CopZ adopts a characteristic β⍺ββ⍺β arrangement, known as a ferredoxin-like fold, in which four-stranded antiparallel β-sheets are flanked by two ⍺-helices (95–97). This ferredoxin-like fold is highly conserved among copper-binding proteins, often referred to as Atx1-like chaperones, named after Atx1 from *Saccharomyces cerevisiae*, the first of its kind to be characterized (98, 99). Interestingly, this fold is not only conserved across Atx1-like proteins but also found in the MBDs of several P_1B_-ATPases, including ATP7A (100), ATP7B (101), and CopA (102).

Another canonical bacterial P_1B_-ATPase is ZntA, first identified in *E. coli* as a major exporter involved in zinc detoxification (103), and later shown to also mediate the efflux of Cd^2+^ and Pb^2+^ (45, 50, 104). Like CopA, ZntA displays the canonical P_1B_-ATPase architecture, with eight transmembrane helices, a conserved CPC motif in TM4, and the three cytosolic A, P, and N domains required for ATP-driven transport (45, 50, 104). ZntA also contains an N-terminal MBD with a ferredoxin-like fold and a CxxC motif that contributes to high-affinity metal coordination (105). Its role in zinc detoxification has since been confirmed in multiple pathogens, including *E. coli* (106), *Salmonella typhimurium* (106, 107), *Vibrio parahaemolyticus* (108), and *Yersinia pestis* (109). These findings underscore the critical role of zinc export systems in pathogen survival and persistence, particularly within host cells, where metal intoxication constitutes an important innate immune strategy.

Although *M. tuberculosis* encodes several P_1B_-ATPases, it does not appear to encode clear homologs of CopA and ZntA. Instead, the primary transporters involved in resistance to metal intoxication differ from these well-characterized systems in several structural and functional aspects, likely reflecting adaptation to the unique intracellular environment encountered by *M. tuberculosis*.

## *M. TUBERCULOSIS* P-TYPE ATPASES: INVENTORY AND FUNCTIONAL SPECIFICITY

**Fig 2:**
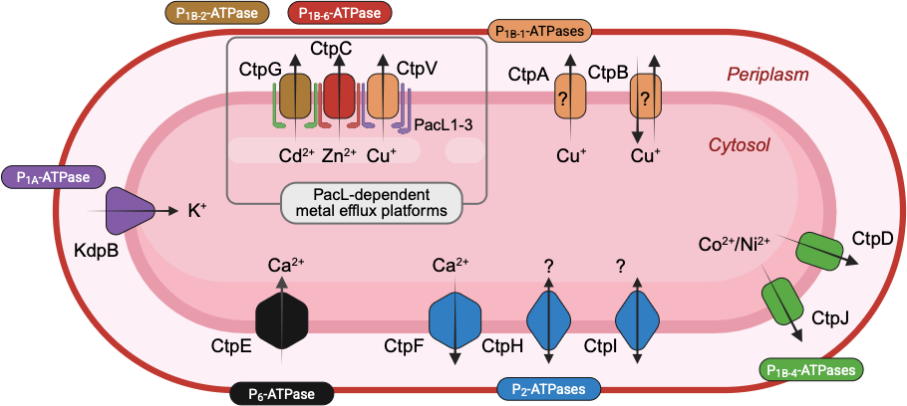
Schematic representation of *M. tuberculosis* P-type ATPases and their substrates. Various P-type ATPases encoded by *M. tuberculosis* are color-coded according to their subfamily classification, based on published experimental data and/or sequence analysis. KdpB (purple) is a P_1A_-ATPase involved in potassium import (110); CtpA and CtpB (orange) are P_1B-1_-copper exporters implicated in the redox stress response (111, 112). CtpD and CtpJ (green) function as cobalt and nickel efflux pumps (52, 55). CtpE (black) and CtpF (blue) are calcium-transporting P_6_- and P_2_- ATPases, respectively (27, 113, 114). CtpH and CtpI (blue) are uncharacterized P_2_-ATPases. CtpC (red) is a zinc-exporting P_1B-6_-like ATPase (13); CtpG (brown) is a P_1B-2_-ATPase responsible for cadmium export (115); and CtpV (orange) is a P_1B-1_-ATPase thought to be involved in copper export (116). Importantly, CtpC, CtpG, and CtpV are co-expressed with their respective PacL scaffold proteins (PacL1, PacL2, and PacL3), forming membrane-associated metal efflux platforms recently termed effluxosomes, which are believed to constitute the primary mechanism of multi-metal resistance in *M. tuberculosis* (117, 118).

Among the 12 P-type ATPases encoded by *M. tuberculosis* (Fig. 2), KdpB is the sole representative of the P_1A_ subfamily, functioning as a potassium importer and forming part of the well-characterized KdpFABC membrane complex (110). The remaining eleven are classified as cation-transporting P-type ATPases (Ctp), designated CtpA through CtpJ and CtpV.

Within this group, CtpF (Rv1997), CtpH (Rv0425c), and CtpI (Rv0107c) belong to the P_2_-type ATPase class. CtpF has been identified as a calcium exporter implicated in *M. tuberculosis* virulence (113, 119). To date, CtpH and CtpI have not been experimentally characterized. However, sequence analysis reveals that both proteins are unusually large (approximately 160 and 170 kDa, respectively) compared with well-known eukaryotic P_2_-type calcium pumps, such as SERCA (110–115 kDa) and SPCA (100–105 kDa). In addition, AlphaFold models suggest that their N-terminal region resembles a degenerate P-type ATPase sequence, whereas their C-terminal region retains the canonical features of a functional P-type ATPase (CtpH: https://alphafold.ebi.ac.uk/entry/AF-P96271-F1, CtpI: https://alphafold.ebi.ac.uk/entry/AF-P9WPS5-F1). Together, these observations suggest that CtpH and CtpI may possess unique functional or regulatory properties. CtpE (Rv0908), previously classified as a P_2_-ATPase, has recently been reassigned to a newly proposed P6 family of P-type ATPases and functions as a calcium importer under calcium-deficient conditions (27, 114).

The remaining seven ATPases, CtpA-D, CtpG, CtpJ, and CtpV, belong to the P_1B_-type ATPase subfamily. Their substrate specificities have been characterized through both experimental studies and sequence-based predictions, particularly by analyzing conserved transmembrane motifs such as CPC or SPC in TM4. These motifs are hallmarks of P_1B-1/2_-ATPases, which typically transport Cu^+^ and Zn^2+^, and P_1B-4_-ATPases, associated with the export of Co^2+^ and Ni^2+^.

CtpA (Rv0092) and CtpB (Rv0103c) are classified as P_1B-1_-ATPases, and both harbor the canonical CPC motif in TM4 as well as a conserved CxxC motif within their N-terminal metal-binding domains, which are hallmarks of copper-transporting ATPases. Functional studies show that CtpA and CtpB exhibit ATPase activity specifically in response to Cu^+^ (but not Cu^2+^) and enhance copper tolerance when expressed in heterologous systems (111, 120). However, *ctpA* or *ctpB* knockout mutants of *M. tuberculosis* do not display significant copper accumulation or increased sensitivity to copper excess, indicating that neither enzyme is essential for primary copper detoxification (111, 112). Unlike classical copper detoxification pumps, *ctpA* and *ctpB* expression is not induced by copper exposure but instead by copper depletion and conditions that mimic the intraphagosomal environment, including hypoxia as well as nitrosative and oxidative stress (111, 112). Notably, both CtpA and CtpB are required for full virulence of *M. tuberculosis* in macrophages and/or mice, and CtpA has been implicated in tolerance to redox stress (112, 121, 122). Together, these observations support the notion that these enzymes contribute to copper homeostasis under host-induced stress conditions and may facilitate copper delivery to periplasmic or membrane-associated proteins involved in stress responses. Intriguingly, a recent study showed that a *ctpB* knockout in *M. tuberculosis* exhibits impaired growth under copper-limited conditions and hypervirulence in the DBA/2 mouse infection model, suggesting a role for CtpB in copper uptake (122).

CtpD (Rv1469) and CtpJ (Rv3743c) are P_1B-4_-ATPases, characterized by the SPC motif in TM4 and implicated in the export of Co^2+^ and Ni^2+^, with some activity also observed for Zn^2+^ (52, 55). The transcription of *ctpJ* is induced by Co^2+^ exposure, and deletion mutants accumulate cobalt, confirming its role in cobalt efflux. CtpD, on the other hand, is upregulated in response to oxidative stress; however, mutants do not exhibit metal accumulation, suggesting a more indirect role in metal homeostasis or possibly cross-regulation with other stress-response pathways (52). More recently, another study has shown that these mycobacterial P_1B-4_-ATPases could play a role in Fe^2+^ homeostasis (54).

Among the 12 P-type ATPases in *M. tuberculosis*, the final three, namely CtpC (Rv3270), CtpG (Rv1992c), and CtpV (Rv0969), have been functionally characterized with respect to their metal substrate specificities. CtpV expression is regulated by CsoR, a copper-sensing transcriptional repressor (123), and its expression is specifically induced upon copper exposure (124). Consistent with this copper-dependent regulation, a ∆*ctpV* mutant of *M. tuberculosis* exhibits reduced survival in the presence of high concentrations of copper in culture (116). Notably, although a Δ*ctpV* mutant is viable in both murine and guinea pig models of TB, it induces reduced lung damage and a dampened immune response, along with prolonged host survival compared to wild-type infection (116). These findings suggest that CtpV mediates copper resistance and contributes to *M. tuberculosis* virulence, likely by enabling the pathogen to persist under host-imposed copper stress. However, ectopic expression of CtpV in *M. smegmatis* does not improve tolerance to either Cu^2+^ or Cu^+^, indicating that this P-type ATPase likely delivers copper to as-yet-unidentified periplasmic enzyme(s) rather than directly mediating detoxification (118).

CtpG is transcriptionally regulated by CmtR, a Cd^2+^-responsive repressor, and is induced by cadmium exposure (125, 126). *In vitro* studies show that CtpG activity is stimulated by several transition metals, including Co^2+^, Zn^2+^, Ni^2+^, Mn^2+^, and Pb^2+^, but its highest substrate specificity is for Cu^2+^ and Cd^2+^ (115). Published studies report divergent conclusions regarding CtpG-mediated metal tolerance in mycobacteria, with evidence supporting tolerance to zinc, cadmium, or copper (115, 127). However, by systematically assessing tolerance to a broad range of metal ions conferred by heterologous expression of *M. tuberculosis* CtpG in *M. smegmatis*, a recent study provides strong evidence that this P-type ATPase specifically confers tolerance to cadmium (118), thereby clarifying previous conflicting reports. To our knowledge, no role for CtpG has been reported in *M. tuberculosis* during infection, but the P-type ATPase was shown to promote the survival of *M. bovis* in THP-1 macrophages and in mice (127).

CtpC functions as a zinc exporter, with expression upregulated in response to zinc exposure through an unknown pathway, and a Δ*ctpC* mutant exhibiting intracellular zinc accumulation and a growth defect under zinc exposure (13). Furthermore, CtpC is involved in intracellular survival within macrophages, consistent with its role in resisting zinc intoxication within the phagosome (13).

To date, no experimental structural data are available for CtpC, CtpG, or CtpV. However, structure prediction tools such as AlphaFold 3 (AF3) suggest that all three proteins adopt a typical P_1B-1/2_-ATPase topology, with eight TM segments, six forming the core membrane domain and two P_1B_-specific auxiliary MA and MB helices, alongside the conserved cytosolic A, P, and N domains, and an N-terminal MBD (CtpC: https://alphafold.ebi.ac.uk/entry/AF-P9WPT5-F1, CtpG: https://alphafold.ebi.ac.uk/entry/AF-P9WPS7-F1, and CtpV: https://alphafold.ebi.ac.uk/entry/AF-P9WPS3-F1). Phylogenetic analysis supports functional assignments consistent with biochemical and genetic data. CtpG clusters with Zn^2+^/Cd^2+^-transporting P_1B-2_-ATPases, in line with its role in cadmium efflux (115, 125), despite having an APC motif in TM4 rather than the canonical CPC motif typical of the P_1B-2_ family, which is nevertheless essential for cadmium transport activity (118). CtpV, harboring a CPC motif, clusters with P_1B-1_-ATPases, consistent with its putative role in copper export and established regulation by the copper-responsive repressor CsoR (116, 123, 124). Surprisingly, CtpC, which also carries a CPC motif and is functionally linked to zinc export, clusters with the poorly characterized P_1B-6_ subfamily. This is supported by the presence of a conserved N-X₁₈-H-N-X-(S/T) motif between TM5 and TM6, a hallmark of P_1B-6_-ATPases, despite its functional similarity to P_1B-2_ proteins (13). Furthermore, CtpC and CtpG MBDs lack the CxxC motif typically associated with metal coordination in copper- and zinc-transporting ATPases. However, previous work has shown that in CopA, the metal-binding capacity of the MBD is not essential for function, suggesting a predominantly regulatory role for these domains (91). Together, these observations indicate that CtpC, CtpG, and CtpV broadly conform to the canonical architecture of P_1B_-ATPases, while retaining unusual features whose functional significance remains to be established.

## PACL PROTEINS: PROTEIN CHAPERONE ROLES

A notable feature shared by CtpC, CtpG, and CtpV is their genetic organization: each is encoded within an operon together with a small membrane protein, now referred to as PacL proteins (for P-type ATPase-associated chaperone-like proteins), with PacL1, PacL2, and PacL3 respectively encoded upstream of CtpC, CtpG, and CtpV (117). These membrane proteins were initially hypothesized to function as metallochaperones, facilitating metal delivery to their cognate ATPases, by analogy with soluble chaperones such as CupA, CopZ, and other Atx1-like proteins (128). However, a key distinction is that CupA, CopZ, and Atx1-like chaperones adopt cupredoxin- or ferredoxin-like folds that are not conserved in PacL proteins. Instead, PacL proteins are defined by the presence of a conserved domain of unknown function (DUF1490), composed of a single transmembrane segment followed by a cytoplasmic region containing several distinct elements, including glutamate/alanine (EA) repeats, an intrinsically disordered region, and an optional C-terminal metal-binding motif (MBM) enriched in histidine residues. A defining feature of PacL-Ctp efflux systems is the strict requirement for PacL proteins to ensure the stability, localization, and/or activity of their cognate P-type ATPases at the plasma membrane. In the absence of PacL1 or PacL2, CtpC or CtpG fail to be stably accumulated, leading to a loss of zinc and cadmium tolerance in *M. tuberculosis* (117, 118). As yet, the role of PacL3 in CtpV activity remains to be investigated.

The chaperone function of PacL proteins is mediated by their direct interaction with the MBD of their cognate P-type ATPases (117, 118). Consequently, PacL1 and PacL2 colocalize with CtpC and CtpG within discrete clusters in the mycobacterial plasma membrane. Further insight into the chaperone function of PacL1 was provided by Boudehen et al. and Dupuy et al., who demonstrated that the EA repeat motifs in the cytosolic regions of PacL1 and PacL2 are essential for binding to the MBDs of CtpC and CtpG and, as a consequence, for the stability of these P-type ATPases (117, 118). However, it remains unclear whether PacL proteins are required for the initial membrane insertion of P-type ATPases or instead for their subsequent stabilization and protection from degradation, as reported for other bacterial metal ion transporters (129, 130)

## PACL PROTEINS: ESSENTIAL SCAFFOLDS FOR EFFLUXOSOMES FORMATION

The three PacL proteins have been shown to interact with one another via a conserved GxxG motif within their transmembrane domain (118). In addition, each PacL protein was shown to interact with the MBDs of CtpC, CtpG, and CtpV. These cross-interactions are essential for the *in vivo* co-localization of CtpC, CtpG, and CtpV together with the three PacL proteins within discrete mycobacterial membrane domains, termed effluxosomes. Within effluxosomes, PacL proteins act as essential membrane scaffolds that display cooperative activities. For instance, they cooperate to stabilize each P-type ATPase component of the effluxosome. CtpC- and CtpG-dependent metal resistance can be restored by co-expression of either of the other PacL proteins in the absence of their cognate PacL chaperone (117, 118). Strikingly, supplementation of the *M. tuberculosis* culture medium with zinc or cadmium, which, respectively, induces the expression of PacL1 or PacL2, influences resistance to the other metal, revealing a cross-resistance mechanism that links zinc and cadmium tolerance in this pathogen. Finally, a recent proximity-labeling approach performed with PacL1 revealed an extensive protein interaction network involving dozens of proteins, suggesting that effluxosomes contribute not only to metal detoxification but also to a broader stress adaptation program (118).

Despite their similarities, some PacL proteins appear to display specific functions within the effluxosome. PacL1 harbors a C-terminal MBM (DLHDHDH), which is absent in PacL2. It was initially shown that a soluble, transmembrane-domain-truncated PacL1 binds zinc in a 1:1 molar ratio, with an apparent K_D_ in the micromolar range (117). However, while this MBM contributes to zinc binding, it is not strictly required for basal zinc resistance. When CtpC is co-expressed with a PacL1 variant lacking the MBM, *M. tuberculosis* retains resistance to 100 µM ZnSO_4_, whereas sensitivity emerges at higher concentrations (250 µM). These observations suggest that PacL1 functions both as an essential structural chaperone and as an accessory metallochaperone, fine-tuning zinc resistance during infection. Strikingly, in the absence of PacL1, PacL2 is able to partially rescue zinc resistance at 100 µM Zn^2+^, although not at 250 µM, unless PacL2 is engineered to include the PacL1 MBM, highlighting the importance of the MBM under conditions of high zinc stress (117). A more recent study showed that PacL1 not only binds zinc but also cadmium and copper, whereas PacL2 does not bind metals (118). This suggests that PacL1 could also actively enhance the activities of CtpG and CtpV and not solely stabilize them. However, whether direct metal transfer occurs between PacL1 and individual P-type ATPases remains to be determined.

Today, it remains unclear what functional advantage is gained by clustering multiple P-type ATPases together. Building on previous research, we recently discussed several examples of bacterial membrane proteins that form clusters, highlighting the potential advantages of such assemblies within membranes (131). These benefits include promoting oligomerization, protecting proteins from degradation, and facilitating preferential subcellular organization. Notably, mutation of the GxxG motif in the transmembrane segment of PacL2 led to a marked reduction in the number of PacL2 clusters, without affecting overall protein abundance (118). Although fluorescence analyses confirmed that CtpG was correctly stabilized and localized to the membrane under these conditions, protein assembly into membrane clusters and cadmium tolerance were both severely impaired, indicating that PacL2-dependent clustering, rather than membrane stabilization alone, is essential for CtpG activity. However, the molecular mechanisms underlying the requirement for clustering in efflux activity remain to be discovered. A compelling mechanistic rationale for the clustering of distinct P-type ATPases emerges from the metal-binding versatility of PacL1. By binding multiple metal species, PacL1 may function as a local metal-buffering hub, concentrating diverse ions within effluxosomes and thereby potentiating the activity of P-type ATPases dedicated to the export of specific metals.

## EFFLUXOSOMES: VERSATILE AND DYNAMIC STRUCTURES

Effluxosomes display a highly dynamic composition that depends on the stresses encountered by *M. tuberculosis*. In particular, environmental metal availability dynamically shapes the composition of PacL-dependent effluxosomes. Dupuy et al. showed that PacL1 forms membrane-associated clusters under basal conditions, whereas PacL2 is weakly expressed and diffusely distributed within the membrane (118). Under metal stress conditions, PacL1 and PacL2 colocalize at the plasma membrane, with zinc promoting more abundant PacL1-enriched clusters and cadmium favoring the formation of PacL2-enriched assemblies. These observations indicate that distinct metal cues selectively remodel effluxosome composition, thereby modulating their architecture and functional specialization.

Using fluorescent protein fusions of PacL1 and CtpC, Boudehen et al. showed that the membrane clusters formed by these two proteins are highly mobile, demonstrating that effluxosomes are not static structures but dynamic hubs for stress sensing and response (118). This behavior is reminiscent of membrane microdomains in eukaryotic cells (132) and may reflect a conserved bacterial strategy to counteract host-imposed metal stress, thereby contributing to microbial resilience and virulence. However, the lipid composition of effluxosomes and the potential influence of specific membrane lipids on their dynamics and metal efflux activity remain unknown. Using super-resolution microscopy coupled with single-particle tracking, Dupuy et al. revealed distinct dynamic populations of PacL2, including highly mobile proteins and more slowly diffusing or immobile molecules likely associated with effluxosomes (118). The observation that individual PacL molecules can transition from a mobile to a confined state supports a model in which PacL proteins dynamically exchange between a diffusible pool and assembled effluxosomes. In light of the metal-binding capacity of some PacL proteins, this mobile fraction may act as membrane “patrollers,” capturing metal ions and delivering them to effluxosomes, or alternatively scanning the membrane for metal-enriched hotspots (133) to nucleate localized effluxosome assembly in response to metal accumulation.

## PACL-ASSOCIATED EFFLUXOSOMES: CONSERVATION, DIVERSITY, AND FUNCTION

PacL proteins define a conserved effluxosome module that extends beyond *M. tuberculosis*. Comparative genomic analyses reveal that PacL proteins are encoded by at least 98 Actinobacteria species, including several pathogenic or opportunistic bacteria, and are invariably associated with P-type ATPase pumps (117). In addition, PacL-like (DUF6110-containing) proteins are encoded across diverse bacterial phyla, as well as in Archaea. Most organisms harbor a single PacL/ATPase pair, but expanded repertoires are observed in some species, such as *Mycobacterium marinum*, which notably encodes five PacL/Ctp pairs. Notably, all identified organisms encode at least one PacL protein with a predicted C-terminal MBM, supporting a conserved metallochaperone function. Altogether, these observations suggest that effluxosome-like assemblies may represent a modular strategy for metal stress management that is conserved across diverse species. Future studies will be required to assess whether effluxosomes function beyond metal detoxification in stress adaptation and to establish whether they contribute to pathogen virulence.

## STRUCTURAL AND EXPERIMENTAL CHALLENGES

One of the main challenges in functionally characterizing the PacL-Ctp efflux systems lies in the fact that PacL proteins are essential scaffolds required for the accumulation of their cognate P-type ATPase at the plasma membrane. On the PacL side, studies with PacL proteins have shown that mutation of the EA repeats in their cytosolic region results in the loss of Ctp(s) membrane localization, indicating that these repeats are critical for protein-protein interactions with P-type ATPases (117, 118). However, dissecting the corresponding interaction interface on Ctp(s) is more complex. If a mutation in the P-type ATPase causes the loss of membrane accumulation, it becomes difficult to determine whether this is due to disruption of the essential PacL-Ctp interaction or simply due to protein misfolding or instability, complicating efforts to functionally interpret the phenotype. As a result, such mutants are unsuitable for purification and structural analysis, as they often yield non-functional or unstable proteins. Given that MBDs in P_1B_-ATPases are now thought to play a regulatory rather than a direct metal-transport role (59, 91), it is plausible that PacL(s) interact with Ctp(s) via their MBD, a possibility supported by interaction assays performed in an ectopic organism (117, 118). This hypothesis opens the possibility of studying the PacL-Ctp MBD interaction *in vitro*, using soluble, recombinant fragments. In principle, this approach is feasible: MBDs with the classical ferredoxin-like fold, such as those from CopA, ZntA, or the soluble chaperones CopZ and Atx1, can be readily expressed and purified in *E. coli* for biochemical and structural studies. However, in the case of CtpC, CtpG, or CtpV, attempts to express their isolated MBDs in standard *E. coli* expression strains (e.g., BL21(DE3)) have yielded highly insoluble protein that aggregates, preventing purification and downstream analysis. This technical limitation presents a significant barrier to dissecting the molecular interface between PacL(s) and Ctp(s) and underscores the need for alternative strategies, such as solubility-enhancing tags, fusion constructs, or heterologous expression systems, for future structural and functional studies.

## CONCLUSION AND FUTURE DIRECTIONS

The ability of *M. tuberculosis* to persist within macrophages, despite sustained exposure to antimicrobial stressors, is central to its pathogenicity and long-term survival. Among the many host-derived defenses it must overcome, metal intoxication by transition elements such as zinc, copper, and potentially cadmium has emerged as a critical component of the immune response (134–136). To counteract this pressure, *M. tuberculosis* has evolved a remarkably diverse set of P-type ATPases, particularly from the P_1B_ subfamily, alongside novel DUF1490-containing scaffold proteins (PacLs) that orchestrate their localization and function. These PacL-Ctp efflux systems enable the bacterium to resist toxic metal accumulation by forming specialized membrane assemblies, which we recently termed effluxosomes, which adaptively respond to distinct metal cues. Importantly, these systems are modular, partially redundant, and subject to complex regulatory inputs, allowing *M. tuberculosis* to fine-tune its detoxification strategy according to the intracellular milieu.

Despite significant progress in elucidating the composition and function of these systems, several open questions remain. Several *M. tuberculosis* P_1B_-ATPases display unusual features relative to canonical bacterial transporters such as CopA and ZntA, including atypical phylogenetic clustering and non-canonical metal-binding motifs. The functional significance of these features remains to be established. Moreover, the essential role of PacL scaffolds in stabilizing and clustering their partner ATPases raises unresolved questions about the molecular nature of PacL-Ctp interactions, particularly given the poor solubility of recombinant MBDs in standard expression systems. Addressing these challenges will require innovative experimental approaches, including advanced protein engineering, co-expression strategies, and structural determination using cryoEM, X-ray crystallography, or NMR.

From a broader perspective, the PacL-Ctp systems of *M. tuberculosis* represent a sophisticated evolutionary solution to the problem of metal intoxication during infection, one that is fundamentally distinct from canonical bacterial efflux systems. Deciphering their structural and regulatory mechanisms not only enhances our understanding of mycobacterial pathogenesis but may also unveil novel therapeutic targets aimed at disrupting metal homeostasis in persistent infections. As such, future work should integrate structural biology, live-cell imaging, and infection models to fully characterize the dynamics and significance of effluxosome platforms in the context of host-pathogen interactions.
